# Shorter Antitubercular Regimens Versus 9 Months of Isoniazid for Latent Tuberculosis in Children: A Systematic Review and Meta-Analysis

**DOI:** 10.1093/cid/ciag073

**Published:** 2026-03-10

**Authors:** Mark Kosenko, Lilit Davtian, Ekaterina Iakovleva, Mukhammad Ashurov, Dmitrii Podgalo, Janna G Oganezova, Elena Kondrikova, Elena Bondarenko, Rita Blandino, Giorgio Sodero, Francesca Raffaelli, Laura Martino, Daniel Munblit, Danilo Buonsenso

**Affiliations:** Department of Paediatrics and Paediatric Infectious Diseases, Institute of Child's Health, Sechenov First Moscow State Medical University, Moscow, Russia; Federal Research and Clinical Center for Children and Adolescents, Federal Medical-Biological Agency of Russia, Moscow, Russia; Department of Paediatrics and Paediatric Infectious Diseases, Institute of Child's Health, Sechenov First Moscow State Medical University, Moscow, Russia; Department of Oncology, The Russian University of Medicine of the Ministry of Health of Russia, Moscow, Russia; Department of Paediatrics and Paediatric Infectious Diseases, Institute of Child's Health, Sechenov First Moscow State Medical University, Moscow, Russia; Academician A.P. Nesterov Department of Ophthalmology of the Institute of Clinical Medicine, Pirogov Russian National Research Medical University, Moscow, Russia; Department of Paediatrics and Paediatric Infectious Diseases, Institute of Child's Health, Sechenov First Moscow State Medical University, Moscow, Russia; Department of Paediatrics and Paediatric Infectious Diseases, Institute of Child's Health, Sechenov First Moscow State Medical University, Moscow, Russia; Department of Woman and Child Health and Public Health, Fondazione Policlinico Universitario A. Gemelli IRCCS, Rome, Italy; Area Pediatrica, Dipartimento di Scienza Della Vita E Sanità Pubblica, Università Cattolica Del Sacro Cuore, Rome, Italy; Pediatric Unit, Azienda Sanitaria Locale di Brindisi, Perrino Hospital, Brindisi, Italy; Pediatric Endocrinology Unit, Perrino Hospital, Brindisi, Italy; Dipartimento Di Scienze Mediche E Chirurgiche, Fondazione Policlinico Universitario Agostino Gemelli IRCCS, UOC Malattie Infettive, Rome, Italy; Department of Woman and Child Health and Public Health, Fondazione Policlinico Universitario A. Gemelli IRCCS, Rome, Italy; Area Pediatrica, Dipartimento di Scienza Della Vita E Sanità Pubblica, Università Cattolica Del Sacro Cuore, Rome, Italy; Department of Paediatrics and Paediatric Infectious Diseases, Institute of Child's Health, Sechenov First Moscow State Medical University, Moscow, Russia; Care for Long Term Conditions Division, Florence Nightingale Faculty of Nursing, Midwifery and Palliative Care, King's College London, London, United Kingdom; Department of Woman and Child Health and Public Health, Fondazione Policlinico Universitario A. Gemelli IRCCS, Rome, Italy; Area Pediatrica, Dipartimento di Scienza Della Vita E Sanità Pubblica, Università Cattolica Del Sacro Cuore, Rome, Italy

**Keywords:** LTBI, shorter LTBI regimen, TB, children

## Abstract

**Background:**

We conducted a systematic review and meta-analysis to compare effectiveness and safety of 9 months of isoniazid (9H) versus shorter rifamycin-containing regimens for treating latent tuberculosis infection (TBI) in children.

**Methods:**

We systematically searched MEDLINE, Embase, and Cochrane Central Register of Controlled Trials to June 2025 for randomized, controlled trials (RCTs) and cohort studies that compared regimens that were shorter than 9 months of isoniazid in children aged 1–18 years. Outcomes were development of TB disease, treatment completion, and adverse events. Risk of bias was assessed using RoB 2.0 and the Risk Of Bias In Non-Randomized Studies - of Interventions (ROBINS-I) tool; certainty of evidence was graded using Grading of Recommendations Assessment, Development, and Evaluation (GRADE).

**Results:**

Five RCTs and 7 nonrandomized studies that enrolled approximately 2950 children in trials and >25 000 in observational cohorts were included. In pooled analysis of 3 RCTs, shorter rifamycin-containing regimens resulted in little to no difference in development of TB disease compared with 9H (odds ratio [OR], 0.19; 95% confidence interval [CI], .03–1.12; moderate-certainty evidence). Treatment completion was probably higher with shorter regimens (OR, 0.51; 95% CI, .42–0.62; moderate-certainty evidence). Adverse events were similar between groups, but evidence is uncertain (low-certainty evidence). Observational data were consistent with these findings, showing higher completion rates and lower hepatotoxicity with shorter treatments.

**Conclusions:**

Shorter rifamycin-containing regimens for pediatric TBI probably increase treatment completion and have similar safety outcomes, with no important difference in development of TB disease compared with the standard regimen. These findings support current guideline recommendations that favor shorter regimens in children.

## Teaser

Shorter rifamycin-containing regimens for pediatric latent tuberculosis infection probably increase treatment completion and have similar safety outcomes, with no important difference in development of tuberculosis (TB) disease compared with the standard 9-month isoniazid (INH) regimen.

Tuberculosis (TB) remains a leading infectious cause of childhood morbidity and mortality, with an estimated 1.2 million children and young adolescents (aged <15 years) developing TB disease and 174 000 deaths occurring in 2024. Despite gradual recovery after the coronavirus disease 2019 pandemic, pediatric case detection and prevention still lag behind targets [1]. Preventing progression from infection to disease is central to the End TB ambitions. Roughly one quarter of the world's population harbors *Mycobacterium tuberculosis* infection; 5%–10% will progress to disease over a lifetime, a risk concentrated in young children and other high-risk groups [2].

For decades, the standard preventive regimen for children with LTBI or TB infection (TBI) has been 9 months of isoniazid (9H) [3]. While efficacious, prolonged treatment compromises completion and carries hepatotoxicity concerns, undermining real-world impact [4]. More recently, the latest World Health Organization (WHO) consolidated guidelines [2] and the WHO operational handbook on the management of TBI in children state that “6 or 9 months of daily isoniazid, or a 3-month regimen of weekly rifapentine plus isoniazid, or a 3-month regimen of daily isoniazid plus rifampicin (RIF)” are recommended options. However, without providing a net preference toward one option over another, these statements are not supported by a methodologically rigorous meta-analysis of pediatric studies that provides a clear number on progression to TB disease and treatment completion based on rigorous definitions.

Indeed, clinicians still face uncertainty when choosing among shorter regimens for children, as indirectly demonstrated by only a slow trend toward adoption of shorter regimens for TBI in both adults and children [5]. Prior reviews often pooled adults with children, lacked stratification by pediatric risk profiles, or did not jointly evaluate the outcomes most relevant to practice, that is, preventing progression to TB disease, maximizing treatment completion, and minimizing adverse events (AEs). In this context, a contemporary, pediatric-focused synthesis that directly compares shorter regimens with 9-month isoniazid is needed to provide more solid evidence to inform guideline implementation and, more importantly, to facilitate and speed program scale-up and translation into real-world settings. Therefore, we conducted a systematic review to compare the effectiveness and safety of antitubercular regimens shorter than 9 months versus the standard 9-month isoniazid therapy in children with TBI, focusing on developing TB disease, treatment completion, and AEs.

## METHODS

This systematic review was conducted in accordance with the Preferred Reporting Items for Systematic Reviews and Meta-Analyses (PRISMA) 2020 statement and the Cochrane Handbook for Systematic Reviews of Interventions. The protocol was registered prospectively with PROSPERO (CRD42025647965).

### Eligibility Criteria

We included randomized, controlled trials (RCTs) and prospective cohort studies that compared any antitubercular regimen shorter than 9 months with the standard 9-month (or longer) isoniazid regimen in children aged 12 months to 18 years with LTBI. We excluded children aged <12 months because they are at very high risk of progressing to active TB and because their management in real-world settings may vary consistently. Also, ruling out active TB in these cases may be particularly challenging. In addition, infants aged <12 months may fall under the umbrella of perinatal TB, being exposed either in utero or soon after delivery (or during delivery).

TBI was defined according to the original trial or study criteria.

Shorter regimens were defined as any course of rifamycin- and/or isoniazid-based therapy of less than 9 months' duration. Studies were eligible if they reported at least 1 of the primary or secondary outcomes outlined below. Retrospective cohorts that met the PICO (patient, population or problem; intervention or interest; comparison or control; outcome) framework were reviewed separately and are summarized in the Supplementary Material. No language or date restrictions were applied.

### Outcomes

The primary outcome was development of TB disease during therapy or after its completion, defined as culture-confirmed *M. tuberculosis* disease and/or TB disease (or active TB) diagnosed using clinical criteria or chest radiography. Secondary outcomes included (1) treatment completion, defined objectively by using directly observed therapy, pill counts, or urine testing; receipt of the prescribed regimen within the intended duration; and applied prespecified thresholds for treatment completion (typically ≥80% of prescribed doses); (2) AEs and serious AEs attributable to therapy; and (3) treatment discontinuation due to AEs.

### Databases and Search Strategy

The search was conducted using MEDLINE and Embase via Ovid and the Cochrane Central Register of Controlled Trials from inception to 19 June 2025. The search strategy combined controlled vocabulary and free-text terms for LTBI, children, isoniazid, rifampicin, rifapentine, and preventive therapy, adapted for each database. Detailed strategies are provided in the Supplementary Material. Reference lists of eligible studies and relevant reviews were hand-searched to identify additional records.

### Study Selection

Pairs of reviewers (L. D., M. A., D. P.) independently screened titles and abstracts for potential eligibility, retrieved full texts of relevant reports, and assessed these against the inclusion criteria using COVIDence systematic review software (Veritas Health Innovation). Any disagreements between the screeners were resolved via consensus or additional reviewer involvement (M. K., D. M.).

Pairs of reviewers (L. D., M. A., D. P.) independently extracted study characteristics, participant demographics, intervention and comparator details, outcome definitions, and numerical results using a piloted standardized form in Microsoft Excel. Discrepancies were resolved by consensus or third-party review. Extracted data included general study features (design, setting, sample size, year, country), participant characteristics (age, sex, comorbidities, LTBI definition), intervention and control regimen details (drug, dose, duration), outcome data (events, denominators, effect measures), and risk-of-bias information.

### Risk-of-Bias Assessment

For RCTs, risk of bias was assessed independently by 2 reviewers (L. D., M. K.) using the Cochrane Risk of Bias tool, 2.0. For nonrandomized studies, the Risk Of Bias In Non-Randomized Studies - of Interventions (ROBINS-I) tool was used. Discrepancies were resolved through consensus or discussion with a third reviewer (E. I., D. M.).

### Certainty of Evidence

We assessed the certainty of evidence for each outcome using the Grading of Recommendations Assessment, Development, and Evaluation (GRADE) framework [6], considering risk of bias, inconsistency, indirectness, imprecision, and publication bias. Evidence was rated as high, moderate, low, or very low certainty.

### Data Synthesis and Statistical Analyses

We planned a narrative synthesis of all included studies, structured by study design, intervention, and outcome. When studies were sufficiently homogeneous in design, population, interventions, and outcomes, we conducted meta-analyses using a random-effects model (DerSimonian–Laird) in Cochrane's Review Manager. For dichotomous outcomes, we calculated pooled odds ratios (ORs) with 95% confidence intervals (CIs). Heterogeneity was assessed using the χ^2^ test (*P* < 0.10 indicating significance) and quantified with the *I*^2^ statistic.

## RESULTS

### Study Selection

**Figure 1.**
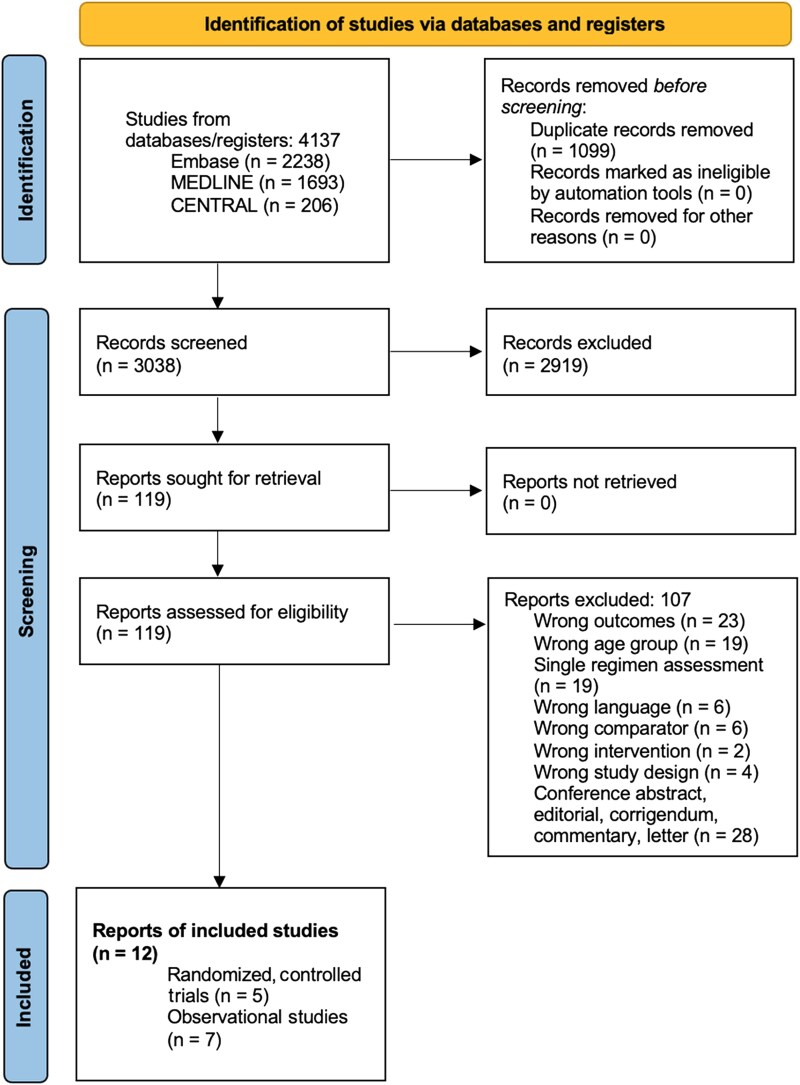
PRISMA (Preferred Reporting Items for Systematic Reviews and Meta-Analyses) flow diagram. Date of last search 19 June 2025. Abbreviation: CENTRAL, Cochrane Central Register of Controlled Trials.

Based on the search strategy, 3038 titles and abstracts were screened for eligibility (Figure 1). Of these, 119 full texts were retrieved for assessment against the inclusion criteria, with 12 studies (reporting results from 11 unique study populations) included in our systematic review.

Five RCTs met the inclusion criteria and are described in Table 1. An additional 7 nonrandomized studies were included and are presented in Table 2. Retrospective cohort studies that otherwise met the PICO framework were reviewed separately and are summarized in the Supplementary Material.

**Table 1.** Characteristics of Randomized, Controlled Trials of 9 Months of Isoniazid Versus Shorter Regimens for Latent Tuberculosis in Children

Author, Year	Design/Setting	Population (Sample Size)	Intervention	Control	Outcome With Available Data	Time Point of Measurement	Overall Risk of Bias (Cochrane Risk of Bias Tool, 2.0)^a^
Apriani et al, 2022	Phase 3, open-label, RCT; single-country substudy of Diallo et al, 2018; Bandung, Indonesia	Children (aged 0–17 y) and adults (aged ≥18 y) with LTBI; 148 children (75 in 4R, 73 in 9H); 855 adults (423 in 4R, 432 in 9H); Eligibility: TST/IGRA-positive with normal chest X ray or stable non-TB abnormalities; excluded if active TB, resistance, pregnancy, drug interactions, or allergy	4 m daily RIF (10–20 mg/kg; max = 600 mg)	9 m daily INH (10–15 mg/kg; max = 300 mg)	Treatment completion ≥80% doses: higher in RIF (84.0% vs 69.9%) TB disease: 1 child (INH, poor adherence) AEs: no grade 3–5 AEs Health system costs: lower with RIF $152.9 vs $206.5	Followed to 16 m post-randomization	Some concerns
Diallo et al, 2018	Multicenter, open-label, noninferiority RCT; Australia, Benin, Brazil, Canada, Ghana, Guinea, Indonesia	844 randomized (422 RIF, 407 INH; MITT, 829) Median age, 10.2 y (IQR, 6.0–13.8); 128 aged <5 years Eligibility: TST >5 mm or QFT + (<1 8 y) and household contacts aged <5 y irrespective of TST results No children with HIV	4 m daily RIF (10–20 mg/kg)	9 m daily INH (10–15 mg/kg)	Treatment completion (≥80% doses): 365/422 (86.5%) RIF vs 314/407 (77.1%) INH; adjusted difference +13.6% (95% CI 7.9–19.3) per protocol analysis completion: 85.3% vs 76.4% TB disease: 0/562 person-years RIF vs 2/542 person-years INH (1 culture-confirmed, 1 NAAT; 1 case after full completion, 1 case in noncompleter; rate difference, 0.37/100 person-years; 95% CI, –.88 to .14) AEs: no drug-related serious AEs; minor AEs approximately 8% visits, no between-group difference	Follow-up 16 m after randomization; TB incidence calculated over approximately 542–562 person-years per arm	Low
Lemvik et al, 2025	Cluster-randomized, open-label, superiority trial; Bandim Health Project, Guinea-Bissau	752 children (aged <15 y) from 223 households exposed to an index TB case INH arm: 354 children (110 clusters) RH arm: 398 children (113 clusters) Mean age, 4.6 y Eligibility: TST >5 mm (aged <15 y) and household contacts aged <5 y irrespective of TST results Children with HIV excluded	4 m daily rifapentine and INH fixed-dose combination (RIF, 15–20 mg/kg; INH, 7.5–10 mg/kg) + pyridoxine	9 m daily INH (10–15 mg/kg; max = 300 mg)	Treatment completion (primary): >80% prescribed pills Completion: INH 68% vs RH 61% (OR, 1.32; 95% CI, .90–1.95) Consecutive completion: INH 40% vs RH 48% (OR, 0.72; 95% CI, .47–1.10) Overall: 57% >80% doses (INH 56% vs RH 58%) TB disease: 1 case in INH arm (12 wk post-enrolment); 0 cases in RH arm Mortality: 1 child in INH group (typhoid fever, unrelated) *AEs:* 258 children reported symptoms (163 INH, 95 RH); 1 RH child had hepatotoxicity (resolved); 1 INH child had seizures; 19 hospitalizations (14 INH, 5 RH), none drug-related	Treatment completion follow-up: through treatment period (3 or 6 m) Clinical follow-up: 2 y post-inclusion (TB morbidity and mortality)	Some concerns
Spyridis et al, 2007	Open-label, RCT; Greece	Children aged <15 y with LTBI, TST-positive, chest X ray normal/inactive lesions, Bacille Calmette–Guérin unvaccinated, immunocompetent; Period 1: group A (n = 232, 9H), group B (n = 238, INH+RIF 4 m)	4 m daily RIF and INH (10 mg/kg each; INH max = 300 mg and RIF max = 600 mg)	9 m daily INH (10 mg/kg; max = 300 mg)	TB disease: no clinical TB cases, but new radiographic changes suggestive of active TB (24% in 9H vs 11.8% in 4 m) → all treated as active TB Treatment completion: Excellent/moderate 86.5% (9H) vs 92.4% (4 m); poor adherence higher in 9H (13.8% vs 7.6%) AEs: No serious AEs; transient liver enzyme elevation (6% in 9H vs 1.2% in short-course); mild gastrointestinal symptoms/skin reactions	Radiographs at baseline, at 4 m (end of short course), and at 1 and 3 y post-treatment; overall follow-up up to 7–11 y (period 1) Treatment completion monitored monthly during treatment with urine strips	High
Villarino et al, 2015	Multicenter, open-label, RCT (nested pediatric cohort of PREVENT TB); 29 sites in United States, Canada, Brazil, Hong Kong, Spain	1058 enrolled children aged 2–17 y (552 RH, 506 INH) MITT: 905 Safety population: 1032 Median age, 11 y (IQR, 4–15) Eligibility: TST >5 mm (aged 5–18 y) and children with high risk for TB HIV-seronegative (aged 2–5 y) and HIV-seropositive (aged 2–18 y) household contacts irrespective of TST results, 5 (<1%) children with HIV	3 m (12 doses) once-weekly rifapentine and INH (RIF: for 10–14 kg, 300 mg; 14.1–25 kg, 450 mg; 25.1–32 kg, 600 mg; 32.1–50 kg, 750 mg and INH: for children aged 2–11 y, 25 mg/kg; for children aged >12 y, 15 mg/kg; max = 900 mg) during directly observed therapy	9 m (270 doses) daily INH (5 mg/kg; aged 2–11 y, 10–15 mg/kg; aged >12 y, 5 mg/kg; max = 300 mg) mostly self-administered, some directly observed therapy	Treatment completion: 415/471 (88.1%) vs 351/434 (80.9%), *P* = .003 Discontinuation due to AE: 8/471 (1.7%) vs 2/434 (0.5%); NS AEs: no treatment-related deaths; grade 3 AEs, 3 (0.6%) vs 1 (0.2%); no grade 4 AEs, no hepatotoxicity Serious AEs: 0 in rifapentine+INH; 7 in INH (2 deaths not related to treatment) TB disease: 0/471 vs 3/434 cases (1 culture-confirmed, 2 clinical) Rates: 0 vs 0.27 per 100 person-years; cumulative risk difference −0.74% (97.5% CI, upper bound +.32%) → met noninferiority margin (0.75%)	Follow-up through 33 m post-enrollment (2320 person-years of observation in MITT)	Some concerns

Abbreviations: 4R, 4 months of daily rifampicin; 9H, 9 months of isoniazid; AE, adverse event; CI, confidence interval; HIV, human immunodeficiency virus; IGRA, interferon gamma releasing assay; INH, isoniazid; IQR, interquartile range; LTBI, latent tuberculosis infection; MITT, modified intention to treat analysis; NAAT, nucleic acid amplification test; NS, not significant; OR, odds ratio; QFT, quantiferon; RH, rifampicin and isoniazid; RCT, randomized, controlled trial; RIF, rifampicin; TB, tuberculosis; TST, tuberculin skin test.

^a^Risk of bias is presented for the prevention of TB disease; detailed domain-level information is available in Figure 2.

**Table 2.** Characteristics of Nonrandomized Studies of 9 Months of Isoniazid Versus Shorter Regimens for Latent Tuberculosis in Children

Author, Year	Design/Setting	Population (Sample Size)	Intervention	Control	Outcome With Available Data	Time Point of Measurement	Overall Risk of Bias Risk Of Bias In Non-Randomized Studies - of Interventions^a^
Gaensbauer et al, 2018	Retrospective cohort, Denver, Colorado, Metro TB Clinic (2006–2015)	1174 children aged <18 y with TBI (395 4R; 779 9H)	4 m (5 d/wk and 7 d/wk regimens, depending on age); RIF (typically targeting 20 mg/kg, within the constraints of available formulations)	9 m (5 d/wk or 7 d/wk regimens, depending on age), INH^b^	Completion: 83.5% (4R) vs 68.8% (9H); AEs rare; no TB cases in either group	Median follow-up approximately 4.5 y	Critical
Cruz and Starke, 2014	Retrospective cohort, Houston, Texas, TB clinic (2010–2013)	404 children (mean age, 7.3 y; 324 on 9H, 80 on 4R)	4 m daily RIF (10–20 mg/kg; max = 600 mg)	9 m twice weekly INH (20–30 mg/kg; max = 900 mg)	Completion: 95% (4R) vs 78% (9H); mild AEs; no TB cases	6–48 m	Serious
Cruz and Starke, 2013	Retrospective cohort, Houston, Texas, TB clinic (1989–2011)	1383 children <18 y; 448 treated for TBI (median age, 2.7 y)	6 m twice weekly RIF (10–15 mg/kg)	9 m twice weekly INH (20–30 mg/kg)	Completion: approximately 95.8%; 2/448 developed TB (0.4%); AEs 6.7%	Median, 1.5 y (up to 23 y)	NA
Ronald et al, 2020	Population-based retrospective administrative database, Quebec, Canada, (2003–2007)	10 559 individuals (mixed age, approximately 2359 aged< 20 y); 9684 on INH; 875 on RIF	4 m (120 doses dispensed over 6 m) RIF^b^	9 m (270 doses dispensed over 12 m) INH^b^	Completion: 53.5% (4R) vs 36.9% (9H); hepatotoxicity lower with RIF; cost lower with RIF; development of TB disease not assessed	Follow-up limited to treatment period (≤12 m)	NA
McNab et al, 2000	Before–after cohort, Canadian Plains Aboriginal communities	994 patients (403 daily INH 12 m; 591 intermittent INH+RIF 6 m); mean age SAP 14.5 y, DOP 8.7 y (includes children)	6 m twice weekly INH (15 mg/kg; max 900 mg/dose) +RIF (10 mg/kg; max = 600 mg), DOT	12 m daily INH self-administered (5 mg/kg; max = 300 mg)	Completion: 82% (6H INH+RIF) vs 19% (12H INH); TB rates 0.9/1000 person-years vs 9/1000 person-years; AEs higher in INH+RIF but not severe	Approximately 6 y	Critical
Kim et al, 2024	Retrospective analysis of national insurance claims, Korea (2016–2020)	11 362 children aged ≤18 y (6463 on 9H; 4899 on 3HR)	3 m daily INH (10 mg/kg; max = 300 mg) + rifampicin (10 mg/kg; max = 600 mg)	9 m daily INH (10–15 mg/kg; max = 300 mg)	Completion: 69.7% (3HR) vs 53.1% (9H); predictors of noncompletion identified; development of TB disease/AEs not assessed	Up to 365 d (9H) or 120 d (3HR)	NA
Rodríguez-Márquez et al, 2025	Before–after cohort, Columbia	…	4 m daily rifampicin (10–20 mg/kg), self-administered and CCS	9 m daily INH (7–15 mg/kg)	Completion: 76.7% (4R) vs 40.4% (9H); 3/213 (1.4%) developed tuberculosis disease in 9H group vs 0/86 in 4R group; the incidence of AEs was lower in the CCS+4R group (3.5%) than in those receiving SOC+9H (11.3%)	24 m (9H) or up to 8 m (4R)	Critical

Abbreviations: 4R, 4 months of daily rifampicin; 6H, 6 months of isoniazid; 9H, 9 months of isoniazid; 12H, 12 months of isoniazid; AE, adverse event; CCS, comprehensive care strategy; CI, confidence interval; DOP, directly observed prophylaxis; DOT, directly observed therapy; HIV, human immunodeficiency virus; IGRA, interferon gamma releasing assay; INH, isoniazid; IQR, interquartile range; LTBI, latent tuberculosis infection; MITT, modified intention to treat analysis; NAAT, nucleic acid amplification test; OR, odds ratio; RH, rifampicin and isoniazid; RCT, randomized, controlled trial; RIF, rifampicin; SAP, self-administered treatment for LTBI; SOC, standard of care; TB, tuberculosis; TST, tuberculin skin test.

^a^Risk of bias is presented for the prevention of active TB; detailed domain-level information is available in Supplementary Figure 4.

^b^Dose is not reported in the article.

The most common reasons for exclusion at the full-text stage were absence of a pediatric population or presentation of data for mixed populations without separate results for children, conference abstracts with no identifiable subsequent publications, and lack of a direct comparison between shorter regimens and the 9-month isoniazid treatment (Supplementary Table 1).

### Clinical Trials

Five RCTs that enrolled children with LTBI were included (Table 1). Three were large, multicenter studies conducted across high-income and low- and middle-income countries [7–9], and 2 were single-country trials [10, 11], with 1 of them being a geographic-specific substudy of a large RCT reported by Diallo et al.

Across all trials, approximately 2950 children were enrolled, with individual study sample sizes ranging from 148 [10] to 1058 [8]. The median age in the largest trials was approximately 10–11 years, but younger children were also represented. Diallo et al included 128 children aged <5 years, and Lemvik et al recruited household contacts with a mean age of 4.6 years. While all of the studies focused on LTBI treatment in clinically/immunologically confirmed cases, 3 of them also included child household contacts aged <5 years regardless of tuberculin skin test (TST) status [7–9]. Children with human immunodeficiency virus (HIV) were excluded in most studies; only Villarino et al included a small number of participants with HIV (<1%).

The interventions evaluated were shorter rifamycin-containing regimens compared with 9 months of isoniazid. These included 4 months of daily rifampicin (4R) [7, 10], 4 months of daily rifampicin plus isoniazid [9, 11], and 12 once-weekly doses of rifapentine plus isoniazid over 3 months [8]. All comparators were 9 months, typically 10–15 mg/kg/d of isoniazid.

Follow-up durations varied from 16 months in Apriani et al and Diallo et al to a maximum of 33 months in Villarino et al

#### Development of TB Disease

All 5 RCTs reported on treatment completion. Four [7–10] assessed incidence of developing TB disease, verified by chest radiographic findings and/or microbiological tests and/or clinical symptoms, as a primary or secondary outcome, while Spyridis reported only new radiographic changes suggestive of active disease.

**Figure 2.**
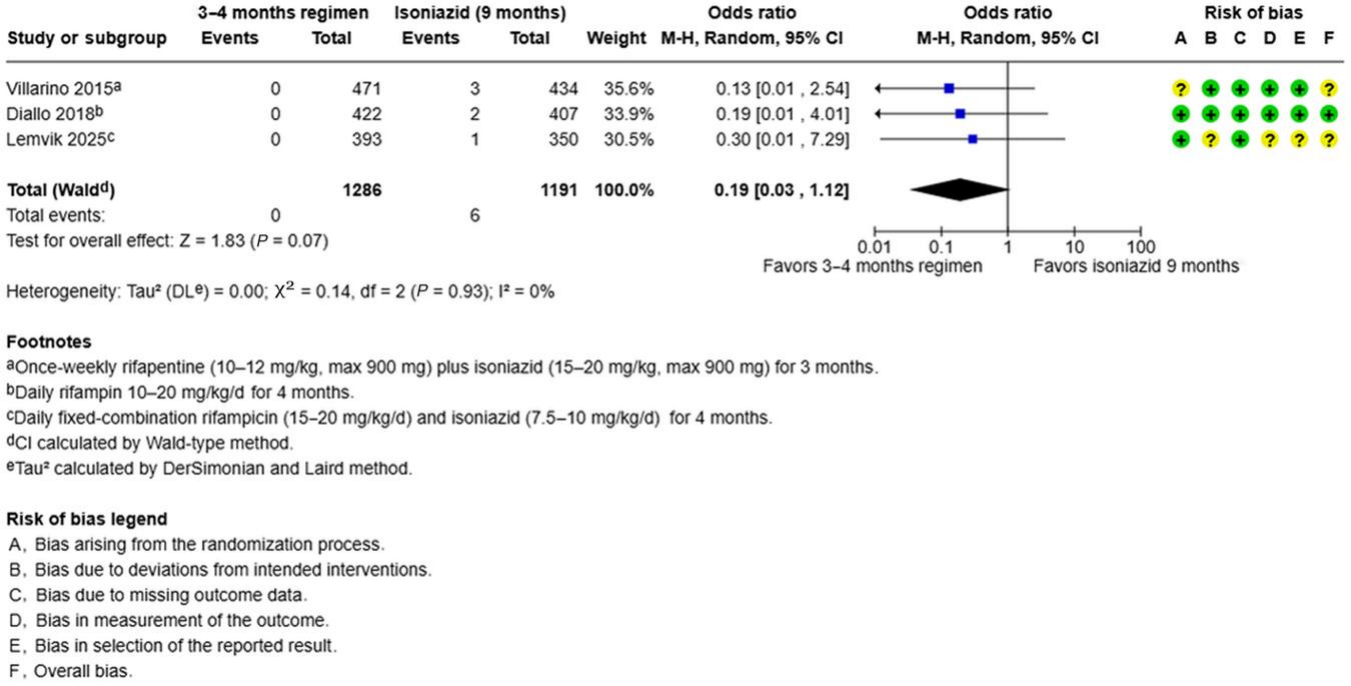
Forest plot for the outcome development of tuberculosis disease after the end of the therapy. Abbreviations: CI, confidence interval; M-H, Mantel-Haenszel.

We conducted a meta-analysis of development of TB disease in children treated with shorter rifamycin-containing regimens (3–4 months) compared with 9 months of isoniazid (Figure 2). Three RCTs contributed data [7–9], with a combined total of 2486 children (1291 in shorter regimens, 1195 in 9-month modality).

**Figure 3.**
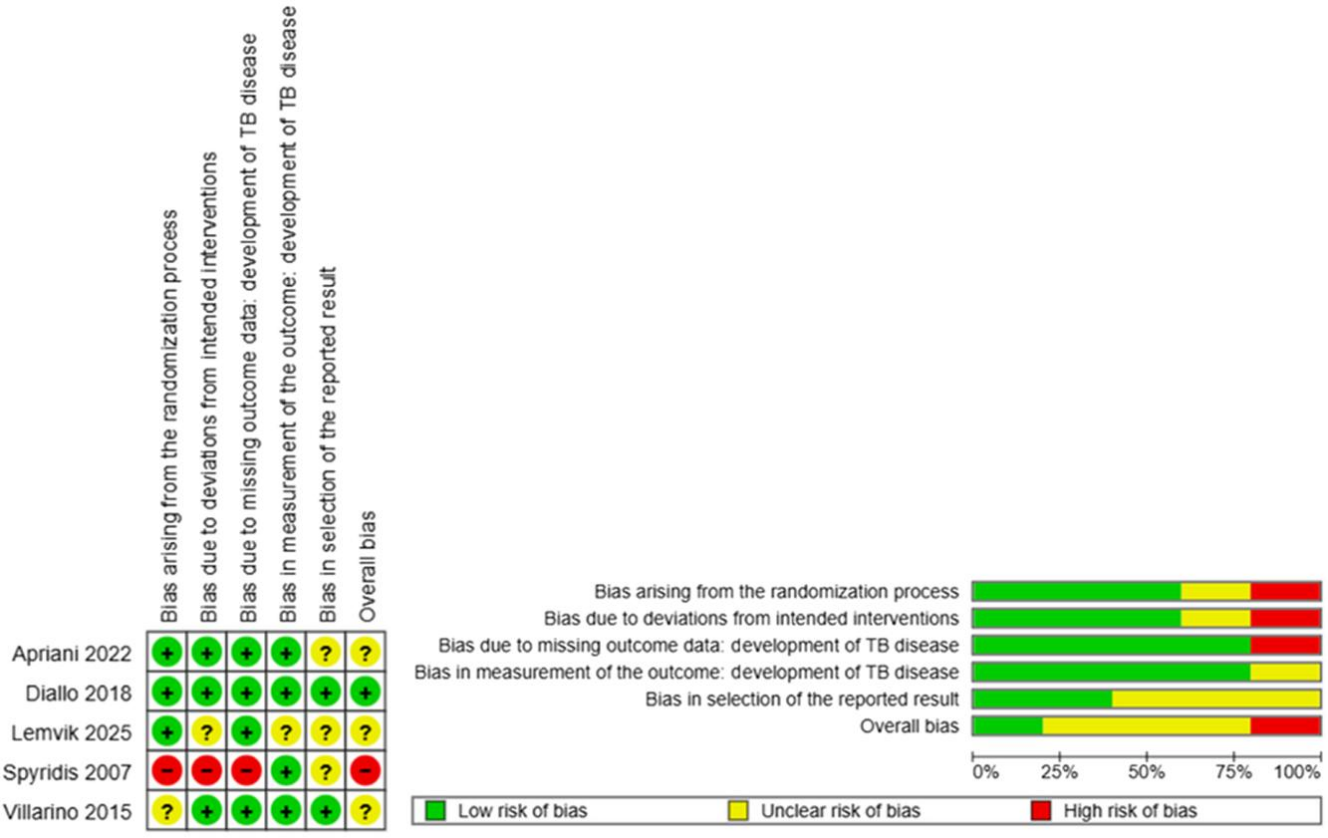
Risk of bias in randomized, controlled trials for the outcome of development of tuberculosis disease after the end of therapy (Cochrane Risk of Bias tool, 2.0).

Across these trials, no children who received shorter regimens developed TB disease, whereas 6 cases were reported in those treated with isoniazid. The pooled OR was 0.19 (95% CI, .03–1.12), suggesting that shorter regimens probably result in little to no difference in development of TB disease compared with 9H. The certainty of this evidence was rated as moderate (Summary of Evidence Table); risk-of-bias assessments indicated overall low to moderate concerns, with most domains judged low risk (Figure 3).

**Summary of Findings 1.** Summary of Findings for Shorter Course Versus Long Course for Latent Tuberculosis in Children

Outcome and Follow-up	Patients (Studies), N	Relative Effect (95% CI)	Absolute Effects (95% CI)			Certainty	What Happens
			Long Course	Shorter Course	Difference		
Development of TB disease	2477 (3 RCTs) [7–9]	OR = 0.19 (.03–1.12)	5 per 1000	1 per 1000 (0–6)	4 fewer per 1000 (from 5 fewer to 1 more)	⨁⨁⨁◯ Moderate^a^	Shorter regimen probably results in little to no difference in development of TB disease
Treatment noncompletion	2947 (4 RCTs) [7–9, 11]	OR = 0.51 (.42–.62)	672 per 1000	511 per 1000 (462–559)	161 fewer per 1000 (from 210 fewer to 113 fewer)	⨁⨁⨁◯ Moderate^b^	Shorter regimen probably results in increase in treatment completion rate
AEs	3403 (4 RCTs) [7–9, 11]	4 trials reported AEs; there is high degree of heterogeneity in AEs reporting across the trials; few events occurred, and generally their frequency was comparable between shorter and longer treatment regimens				⨁⨁◯◯ Low^c^	Shorter regimen may result in little to no difference in AEs compared with longer, but the evidence is uncertain

Abbreviations: AE, adverse event; CI, confidence interval; OR, odds ratio; RCT, randomized, controlled trial; TB, tuberculosis.

^a^Downgraded 1 level for imprecision (the 95% CI includes appreciable benefit and no effect).

^b^Downgraded by 1 level for serious risk of bias: Spyridis et al [11] was assessed as at high risk of bias with some concerns regarding randomization and deviations from the intended interventions and Villarino et al [8] for some concerns regarding outcome measurement.

^c^Downgraded by 2 levels for serious risk of bias in measurement of the outcome and selection of the reported result and substantial heterogeneity in AE reporting across trials.

#### Treatment Completion

**Figure 4.**
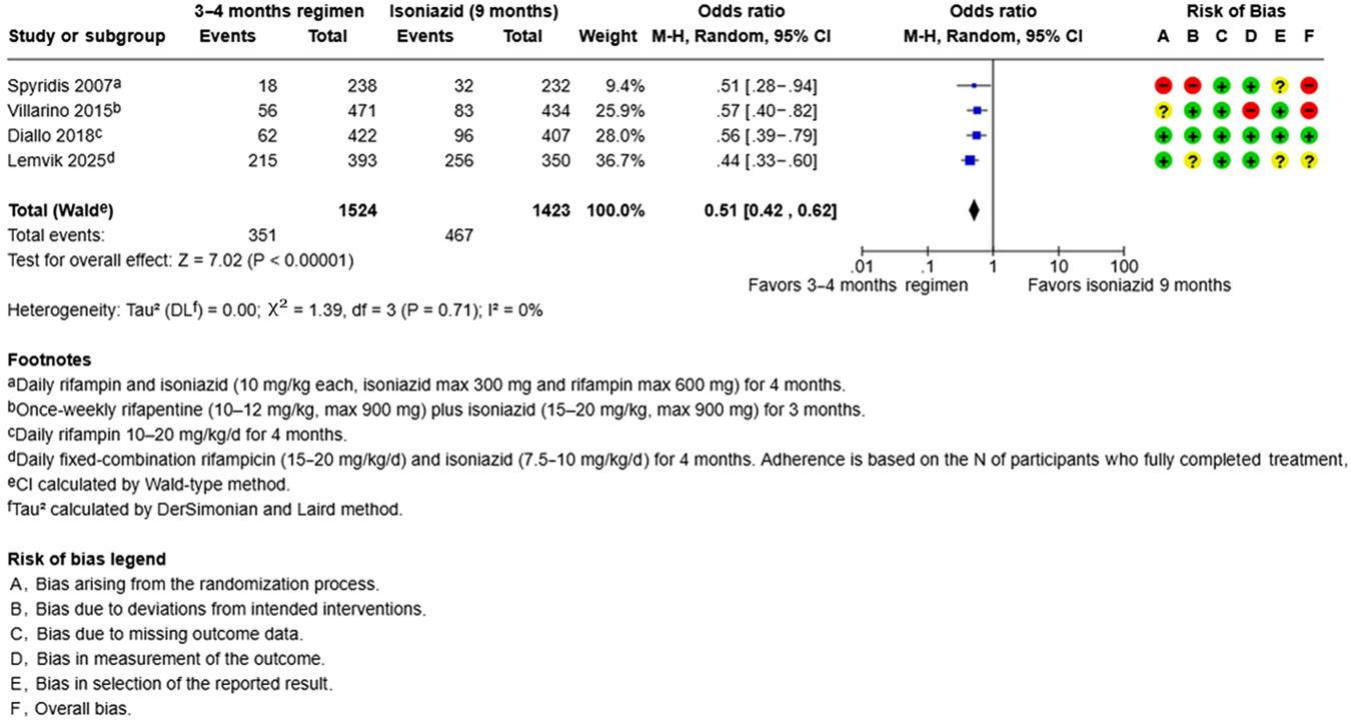
Forest plot for the outcome of rates of treatment completion. Number of events in each group equals number of trial participants who did not complete the treatment modality. Abbreviations: CI, confidence interval; M-H, Mantel-Haenszel.

Four RCTs, including a total of 2947 children, compared rates of treatment completion to shorter rifamycin-containing regimens with the standard 9-month isoniazid regimen (Figure 4). All studies assessed treatment completion objectively, as mentioned in the Methods section.

Across studies, the proportion of children who completed treatment ranged from 45% to 93% for short rifamycin-based regimens and from 27% to 86% for 9-month isoniazid. In every trial, completion rates were higher with the shorter regimen.

The pooled random-effects meta-analysis showed that shorter regimens increase the likelihood of treatment completion compared with 9-month isoniazid (OR, 0.51; 95% CI, .42–.62). The direction and magnitude of effect were consistent across all included trials. The certainty of evidence was rated moderate, downgraded 1 level for high risk of bias in the trial by Spyridis et al due to the quasi-randomized design and exclusion of participants with poor adherence from the analysis and high risk of bias in measurement of the outcome in the trial by Villarino et al due to [8, 11] (Supplementary Figure 1).

#### Adverse Events

AEs and tolerability were consistently monitored, with hepatotoxicity and other severe events rare across studies. No treatment-attributed deaths were reported in any trial. Serious AEs attributed to treatment were not reported in any of the trials. Serious AEs not attributed to treatment were consistently lower with shorter regimens (Villarino et al [8], 0 of 539 versus 7 of 493 in 9H, and Lemvik et al [9], 5 of 393 versus 14 of 350). Treatment discontinuation due to AEs was similar across all trials (Supplementary Figure 2).

Across the included trials, shorter regimens may result in little to no difference in the frequency of AEs compared with the 9-month isoniazid regimen. Minor AEs were generally less common with shorter courses. In Diallo et al [7], 5.1% of participants who received a shorter regimen reported at least 1 minor symptom versus 13.6% with 9H; in Lemvik et al [9], it was 24.2% versus 46.6% (Supplementary Table 2). Specific events such as transient rash or photosensitivity were infrequent, leading to imprecise estimates.

Hepatotoxicity was rare across all studies; only 1 case was reported in a shorter-regimen arm, with none in the 9H groups. Transient elevations in liver enzymes were uncommon and appeared less frequently with shorter regimens, although the small number of events limits confidence in this finding. The certainty of evidence was rated as low due to serious risk of bias that arose primarily from sections “bias in measurement of the outcome” and “bias in selection of the reported result” (Supplementary Figure 3), as well as substantial heterogeneity in AE reporting across trials.

### Nonrandomized Studies

Seven nonrandomized studies were included in the systematic review (Table 2). Four studies were conducted in North America [12–15], 1 in Canada's Aboriginal communities [16], 1 in Korea [17], and 1 in Colombia [18]. Most were retrospective cohorts from high-income settings, with 1 population-based administrative database analysis and 2 before–after community-based studies. Sample sizes varied considerably, from a few hundred children in single-center clinic cohorts [13, 14] to more than 11 000 children in a national insurance database [17].

Together, these studies included more than 25 000 children and adolescents with LTBI. Mean or median ages ranged widely, from 2.7 years in the Houston cohort [14] to mid-adolescence in the Aboriginal before–after study [16]. HIV status was rarely reported, and diagnostic criteria for LTBI varied across settings.

The regimens assessed included 4 months of daily rifampicin [12, 13, 15, 18], 3 months of daily isoniazid plus rifampicin [17], and 6 months of intermittent isoniazid–rifampicin combination under directly observed therapy [16]. Comparators were usually 9 months of daily isoniazid, though some used longer isoniazid regimens or intermittent dosing schedules [16].

Development of TB disease was assessed in 4 studies. Three cases of active TB were reported in the study by Rodríguez-Márquez et ; all of the participants received 9 months of isoniazid (0.9% of treated children in the 9INH group). TST and interferon gamma releasing assays (IGRAs) of 1 child from the same regimen group were unknown. Two cases were reported in Cruz and Starke [14] (0.4% of treated children in both groups). McNab et al [16] observed markedly lower TB rates with intermittent INH+RIF compared with prolonged isoniazid, although development of TB disease was not eliminated. In contrast, Gaensbauer et al [12] and Cruz and Starke [13] reported no cases during follow-up. The remaining 3 studies [13, 15, 17] did not assess development of TB disease.

Treatment completion was consistently higher with shorter regimens, ranging from 77%–96% with a 3–4 month regimen to 40%–79% with 9H. The largest national insurance claims study from Korea similarly reported completion rates of 70% for 3 months compared with 53% for 9H.

AEs were infrequent. When reported, hepatotoxicity was lower in rifampicin-based regimens [15], and serious AEs were rare [12, 16].

Of the 7 studies, 4 [12, 13, 16, 18] reported results on development of TB disease. The risk of bias for this outcome was high, with 3 studies rated as critical and 1 as serious (Supplementary Material). All 7 studies were assessed for risk of bias regarding treatment completion, with 6 at a critical risk and 1 at a serious risk (Supplementary Figures 4 and 5). These judgments were primarily due to potential baseline and time-varying confounding and deviations from intended interventions, the latter stemming from the use of different therapy administration regimens (direct observed therapy versus self-administered therapy).

## DISCUSSION

In this systematic review, we synthesize evidence from 5 RCTs and 6 nonrandomized studies to evaluate antitubercular regimens shorter than 9 months compared with the standard 9-month isoniazid regimen in children with LTBI. The findings indicate that shorter rifamycin-containing regimens probably increase treatment completion and probably result in little to no difference in development of TB disease, with low-certainty evidence suggesting similar or fewer AEs.

The consistency of these findings across trial and observational settings strengthens confidence in the robustness of shorter regimens for treatment of TB infection in children. In our pooled analysis of development of TB disease, no events occurred in children treated with 3- to 4-month rifamycin-based regimens compared with 6 events in those who received longer treatment. TB disease was defined as either microbiologically confirmed disease or by clinical diagnosis (probable TB). Despite this heterogenicity of the outcome definition (which is consistent with real-world settings as TB disease is frequently paucibacillary in children) and although this difference did not reach statistical significance due to the very small number of events, the direction of effect is consistent across the included studies and also with adult evidence [4, 8, 19, 20].

Treatment completion was consistently higher with shorter regimens in 4 of the 5 included RCTs. Under trial conditions, completion rates with shorter regimens exceeded 84% compared with 70%–81% for 9-month treatment. Only the cluster-randomized trial in Guinea-Bissau failed to show a completion advantage, with poor outcomes in both groups. This finding is likely related to household mobility and fragile program infrastructure [9], which can reflect a more realistic real-world scenario compared with RCTs (at least in low-income settings). Nonrandomized studies reinforced these results, reporting substantially higher completion with 4R or 3HR in large program datasets, including a Korean national insurance study of more than 11 000 children in which shorter regimen completion reached 70% versus 53% with 9-month modality [17]. These findings echo programmatic reports in adults and children who identify long treatment duration as the principal barrier to completion.

AEs were uncommon across all study designs, and low-certainty evidence suggests that shorter regimens may result in little to no difference in the frequency of AEs compared with 9H. Hepatotoxicity was rare and generally less frequent with rifamycin-containing regimens. No treatment-related deaths were reported. Of note, when assessed, shorter regimens were also found to be consistently cheaper in both clinical trials and real-world settings [4, 7, 15, 21], although a cost analysis was not an outcome of our meta-analysis.

Our findings align with the evolving international policy landscape, suggesting that even in active TB, shorter regimens are frequently effective and safe in both adults and children [22, 23]. Our meta-analysis, which included the most recent updates, further reinforces this direction for the pediatric population infected with TB, representing potential advantages not only in terms of treatment completion and safety outcomes but also in terms of reducing potential drug interactions in real-world settings. Traditional 9-month INH therapy may remain an option for children who cannot tolerate RIF-based regimens.

Program realities remain a challenge. Failure to identify and initiate children on preventive therapy and barriers such as drug formulation or availability of rifapentine are key obstacles [24, 25].

Our systematic review has several strengths including being a comprehensive search, inclusion of both trial and observational data, and application of GRADE to assess certainty of evidence. Limitations include the small absolute number of pediatric TB disease development events, heterogeneity in outcome definitions (eg, radiographic end points in older studies), and exclusion of children with HIV from most trials. It is important to clarify that some studies [7–9] included pediatric household contacts (aged <5 years) regardless of TST/IGRA status. Our team discussed whether to include the particular population of young children with high-risk adult contacts with TB. We decided to include these children independently from TST/IGRA because in real-world settings these children would require preventive therapy (if active disease is excluded). Therefore, we concluded that it is important to consider the relative effectiveness of standard versus shorter therapies in this specific population, despite the fact that they could not be strictly considered as having LTBI. This clarification is also important because a positive TST/IGRA in children aged <5 years with close adult contacts with TB is a significant predictor for TB progression compared with children with a negative TST/IGRA, if not treated [26]. Despite these differences, we believe that our findings suggest shorter preventive therapies in this population. Last, data from Spyridis et al [11] need to be carefully considered, given the unusually high levels of possible new changes suggestive of TB disease in children treated with standard or shorter regimens (despite numbers being significantly smaller in the shorter-regimen groups). Importantly, these children were asymptomatic, and even the authors discussed that interpretation of chest radiographs is subjective and may be difficult to distinguish between infection and disease.

In conclusion, shorter rifamycin-containing regimens, particularly 4R and 3HP, probably achieve higher completion rates and safety outcomes compared with those for 9H, with no evidence of reduced protection against disease progression. These findings reflect a transition toward shorter, safer, and more feasible preventive regimens for children, representing an important step toward achieving End TB targets through effective primary prevention. Future research should focus on younger children, children with HIV, and implementation strategies in high-burden, resource-limited settings where system-level barriers may constrain the benefits of shorter therapy.

## Supplementary Material

ciag073_Supplementary_Data

## Supplementary Data

Supplementary materials are available at *Clinical Infectious Diseases* online. Consisting of data provided by the authors to benefit the reader, the posted materials are not copyedited and are the sole responsibility of the authors, so questions or comments should be addressed to the corresponding author.
