# Supplementary material for: Shorter Antitubercular Regimens Versus 9 Months of Isoniazid for Latent Tuberculosis in Children: A Systematic Review and Meta-Analysis
Source: Clin Infect Dis. 2026 Mar 10;83(1):e155–66. doi: 10.1093/cid/ciag073 (PMC13393115; doi:10.1093/cid/ciag073)
Supplement: ciag073_Supplementary_Data [file ciag073_supplementary_data.zip › Supplementary material_14.01.2026.docx]

**Supplementary material**

**Shorter antitubercular regimens versus 9-month isoniazid for latent tuberculosis in children: A Systematic Review and meta-analysis**

Mark Kosenko^1^*, Lilit Davtian^2^*, Ekaterina Iakovleva^1^*, Mukhammad Ashurov^3^*, Dmitrii Podgalo^1^*, Janna G. Oganezova^4^, Elena Kondrikova^1^, Elena Bondarenko^1^, Rita Blandino^5,6^, Giorgio Sodero^7,8^, Laura Martino^5,6^, Daniel Munblit^1,9^*, Danilo Buonsenso^5,6^*

1. Department of Paediatrics and Paediatric Infectious Diseases, Institute of Child’s Health, Sechenov First Moscow State Medical University, Moscow, Russia
2. Federal Research and Clinical Center for Children and Adolescents, Moscow, Russia
3. FSBEI of MOH of Russia, Moscow, Russia
4. Academician A.P. Nesterov Department of Ophthalmology of the Institute of Clinical Medicine, Pirogov Russian National Research Medical University, Russia
5. Department of Woman and Child Health and Public Health, Fondazione Policlinico Universitario A. Gemelli IRCCS, Rome, Italy
6. Area Pediatrica, Dipartimento di Scienza Della Vita E Sanità Pubblica, Università Cattolica Del Sacro Cuore, Roma, Italy
7. Pediatric Unit, Azienda Sanitaria Locale di Brindisi, Perrino Hospital, Italy
8. Pediatric Endocrinology Unit, Perrino Hospital, Brindisi, Italy
9. Care for Long Term Conditions Division, Florence Nightingale Faculty of Nursing, Midwifery and Palliative Care, King’s College London, London, UK

# **Search strategies**

**Database: Embase Classic+Embase <1947 to 2025 June 19> (via Ovid)**

1. exp child/

2. exp infant/

3. exp adolescent/

4. exp pediatrics/

5. "toddler*".ab,ti.

6. "paediatri*".ab,ti.

7. "pediatri*".ab,ti.

8. baby.ab,ti.

9. babies.ab,ti.

10. "neonat*".ab,ti.

11. "newborn*".ab,ti.

12. "new born*".ab,ti.

13. "girl*".ab,ti.

14. "boy*".ab,ti.

15. (kindergarten* or preschool* or school*).ab,ti.

16. "teen*".ab,ti.

17. "youth*".ab,ti.

18. "juvenile*".ab,ti.

19. (young adj (person or people)).ab,ti.

20. "minors*".ab,ti.

21. 1 or 2 or 3 or 4 or 5 or 6 or 7 or 8 or 9 or 10 or 11 or 12 or 13 or 14 or 15 or 16 or 17 or 18 or 19 or 20

22. latent tuberculosis/

23. latent tuberculosis.ab,kf,ti.

24. 22 or 23

25. 21 and 24

**Ovid MEDLINE(R) Epub Ahead of Print, In-Process & Other Non-Indexed Citations, Ovid MEDLINE(R) Daily and Ovid MEDLINE(R) <1946 to June 19, 2025 >**

1. exp child/

2. exp infant/

3. exp adolescent/

4. exp pediatrics/

5. "toddler*".ab,ti.

6. "paediatri*".ab,ti.

7. "pediatri*".ab,ti.

8. baby.ab,ti.

9. babies.ab,ti.

10. "neonat*".ab,ti.

11. "newborn*".ab,ti.

12. "new born*".ab,ti.

13. "girl*".ab,ti.

14. "boy*".ab,ti.

15. (kindergarten* or preschool* or school*).ab,ti.

16. "teen*".ab,ti.

17. "youth*".ab,ti.

18. "juvenile*".ab,ti.

19. (young adj (person or people)).ab,ti.

20. "minors*".ab,ti.

21. 1 or 2 or 3 or 4 or 5 or 6 or 7 or 8 or 9 or 10 or 11 or 12 or 13 or 14 or 15 or 16 or 17 or 18 or 19 or 20

22. latent tuberculosis.ab,kf,ti.

23. Latent Tuberculosis/

24. 22 or 23

25. 21 and 24

**Cochrane Controlled Register of Trials (CENTRAL)**

Trials matching MeSH descriptor: [Latent Tuberculosis] explode all trees


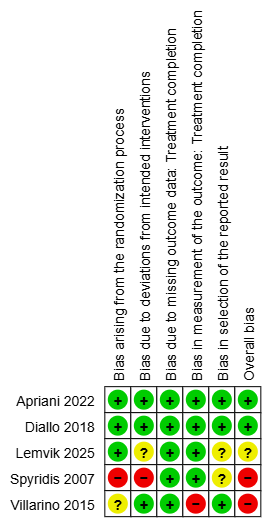


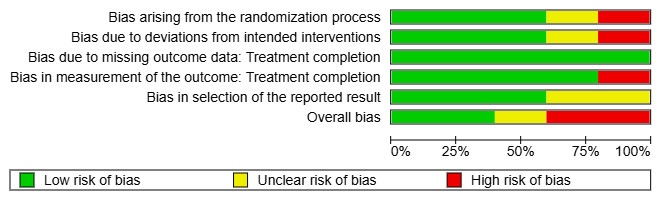


**Figure S1.** Risk of bias in randomized controlled trials for the outcome treatment completion (Cochrane RoB 2.0).

**Figure S2.** Treatment discontinuation due to adverse events in randomised controlled trials comparing shorter rifamycin-containing regimens with 9-month isoniazid in children with latent tuberculosis infection. No pooled estimate is provided due to high heterogeneity of outcome assessment.


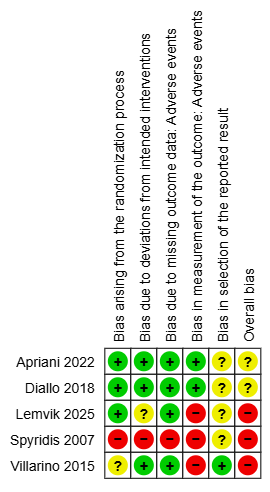

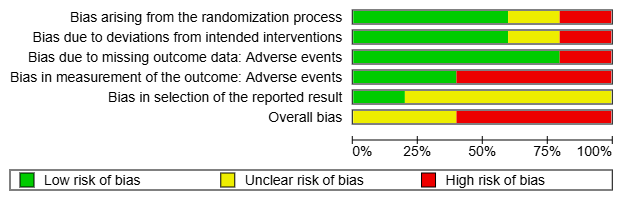


**Figure S3.** Risk of bias in randomized controlled trials for the outcome Adverse events (Cochrane RoB 2.0).


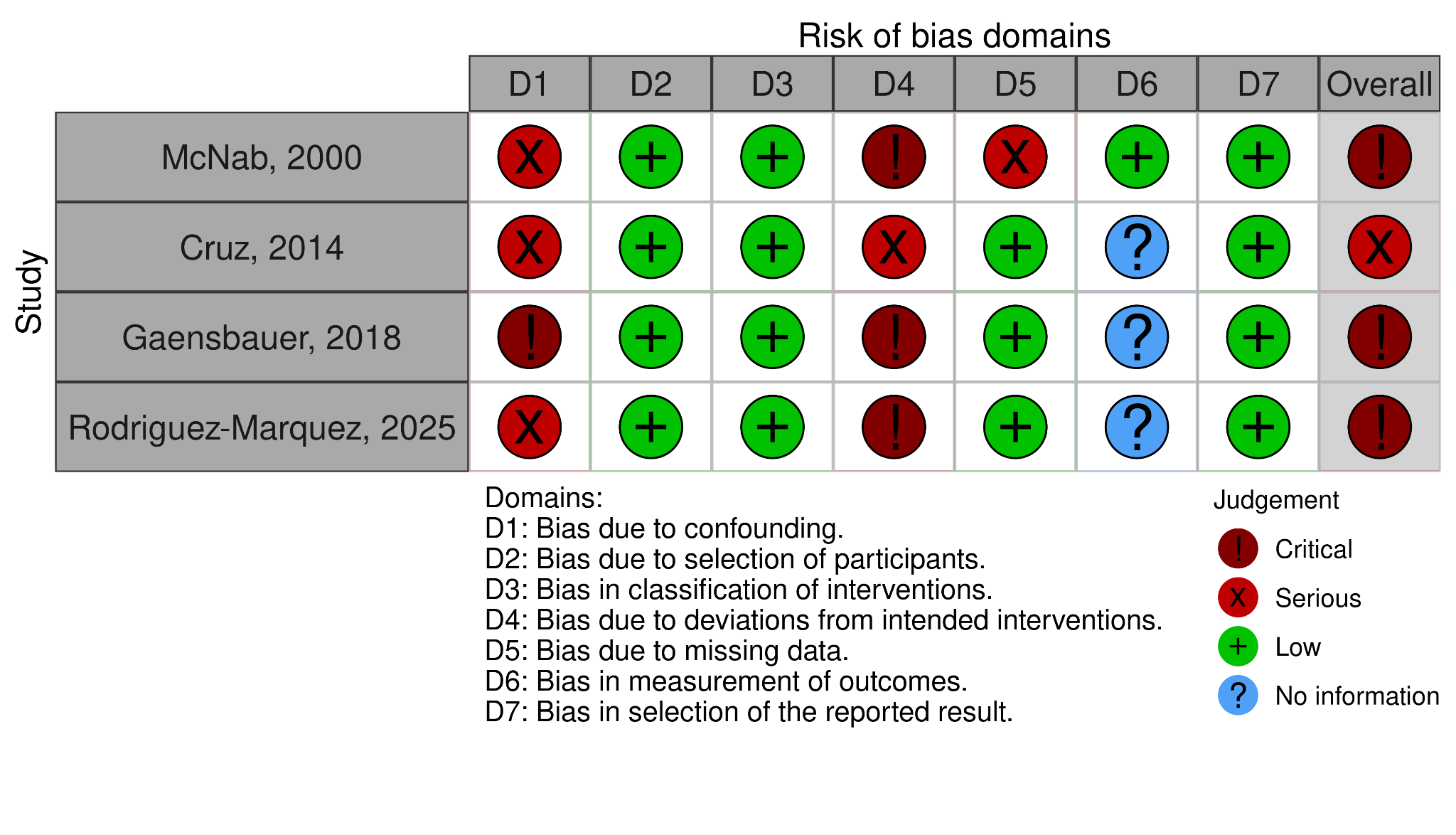


**Figure S4.** Risk of bias in non-randomized studies of interventions (ROBINS-I) for the outcome development of tuberculosis disease.


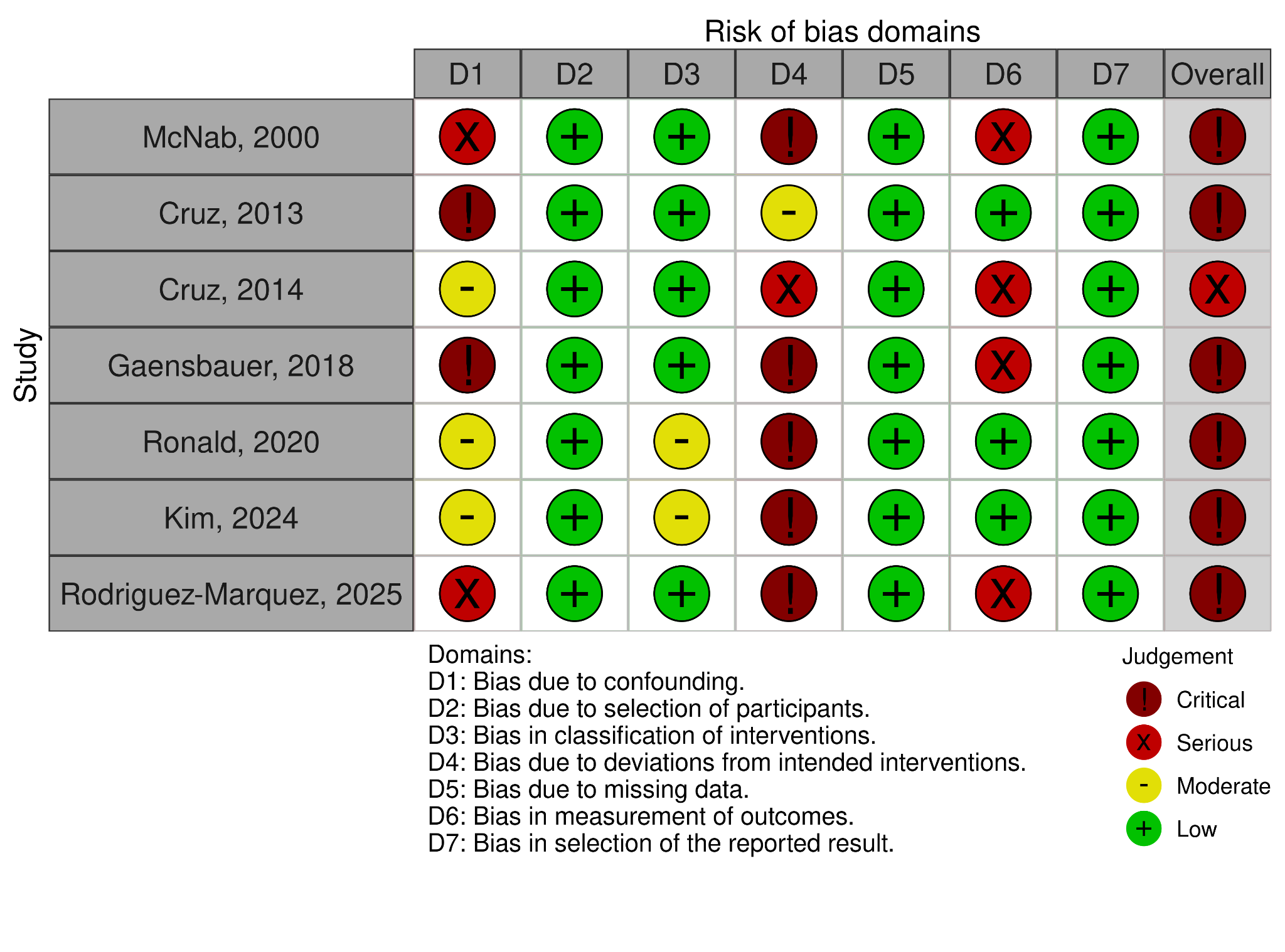


**Figure S5.** Risk of bias in non-randomized studies of interventions (ROBINS-I) for the outcome treatment completion.

| **Study** | **Title** | **Exclusion reason** |
| --- | --- | --- |
| Adepoju 2023 | **Completion of 6-mo isoniazid preventive treatment among eligible under six children: A cross-sectional study, Lagos, Nigeria** | Wrong comparator: no data on longer regimens |
| Adetifa 2013 | **Interferon-γ ELISPOT as a biomarker of treatment efficacy in latent tuberculosis infection: a clinical trial** | Single regimen assessment |
| Agca 2016 | **Preventive treatment for latent tuberculosis infection** | Conference abstract |
| Ahmad 2020 | **Latent tuberculosis infection among minor asylum seekers in Denmark** | Commentary |
| Aki 2018 | **The incidence of tuberculosis infection in hematopoietic stem cell transplantation recipients: A retrospective cohort study from a center in Turkey** | Data on TB incidence in specific population |
| Alashker 2018 | **Treatment outcomes of occult tuberculosis in dialysis patients** | Wrong age group |
| Albanna 2013 | **Serum lipids as biomarkers for therapeutic monitoring of latent tuberculosis infection.** | Letter to the Editor |
| Alvarez 2020 | **The implementation of rifapentine and isoniazid (3HP) in two remote Arctic communities with a predominantly Inuit population, the Taima TB 3HP study** | Mixed data for children and adults |
| AmbronadeMarcos 2018 | **Compliance of latent tuberculosis infection treatment in a cohort of TB contacts** | Wrong language |
| Anger 2012 | **Active case finding and prevention of tuberculosis among a cohort of contacts exposed to infectious tuberculosis cases in New York City.** | Single regimen assessment |
| Anonymous 2010 | **Severe isoniazid-associated liver injuries among persons being treated for latent tuberculosis infection - United States, 2004-2008** | Wrong study design: case-series |
| Aspler 2010 | **Impact of treatment completion, intolerance and adverse events on health system costs in a randomised trial of 4 months rifampin or 9 months isoniazid for latent TB** | Wrong age group |
| Bamrah 2014 | **Treatment for LTBI in contacts of MDR-TB patients, Federated States of Micronesia, 2009-2012** | Single regimen assessment for MDR tuberculosis |
| BarbosaSilva 2016 | **Non-completion of latent tuberculous infection treatment among children in Rio de Janeiro State, Brazil** | Wrong outcomes: mixed data for 6 and 9 months of isoniazid |
| Bastos 2013 | **Changes in QuantiFERON®-TB Gold In-Tube results during treatment for tuberculous infection** | Wrong age group |
| Bastos 2020 | **A public health approach to increase treatment of latent TB among household contacts in Brazil** | Health systems interventions |
| Bastos 2020 | **Health system costs of treating latent tuberculosis infection with four months of rifampin versus nine months of isoniazid in different settings** | Wrong outcomes: costs analysis |
| Bauer 2012 | **Health-related quality of life in persons treated for active and latent tuberculosis, versus persons screened but not treated for tuberculosis, from diagnosis through two months of follow-up** | Conference abstract |
| Benjumea-Bedoya 2023 | **Implementation of an integrated care strategy for child contacts of tuberculosis patients: a quasi-experimental study protocol** | Study protocol |
| Bennet 2004 | **Positive tuberculin reaction in children: from a life insurance to a ticking bomb. Latent tuberculosis should be treated to reduce the risk of active disease** | Wrong language |
| Berrocal-Almanza 2022 | **Effectiveness of nationwide programmatic testing and treatment for latent tuberculosis infection in migrants in England: a retrospective, population-based cohort study** | Mixed data for children and adults |
| Bishara 2014 | **[Accessibility and compliance of Ethiopian immigrants to latent tuberculosis infection treatment]** | Wrong language |
| Bonnet 2017 | **Prospective cohort study of the feasibility and yield of household child tuberculosis contact screening in Uganda** | Single regimen assessment |
| Boyd 2017 | **Treatment of latent tuberculosis infections in the darwin region** | Short Report |
| Bright-Thomas 2010 | **Effectiveness of 3 months of rifampicin and isoniazid chemoprophylaxis for the treatment of latent tuberculosis infection in children** | Single regimen assessment |
| Burman 2021 | **Is the treatment of latent tuberculosis infection amongst recent migrants safe and effective in primary care?** | Conference abstract |
| Burman 2024 | **Treatment of latent tuberculosis infection in migrants in primary care versus secondary care.** | Wrong age group |
| CabralMendonca 2016 | **Abandonment of treatment for latent tuberculosis infection and socioeconomic factors in children and adolescents: Rio de Janeiro, Brazil** | Single regimen assessment |
| Cakar 2018 | **Evaluation of treatment indications, tuberculin skin test, and bacillus calmette-guerin vaccination scars in the cases of latent tuberculosis infection treatment** | Wrong outcomes: mixed data for 6 and 9 months of isoniazid |
| Campbell 2023 | **Multicenter Analysis of Attrition from the Pediatric Tuberculosis Infection Care Cascade in Boston** | Wrong outcomes: mixed data for active and latent TB |
| Cass 2005 | **Structured behavioral intervention to increase children's adherence to treatment for latent tuberculosis infection** | Wrong intervention |
| Catano 2015 | **Follow-up results of isoniazid chemoprophylaxis during biological therapy in Colombia** | Wrong age group |
| Chan 2017 | **Safety and tolerability for once-weekly Rifapentine plus Isoniazid in Taiwan** | Conference abstract |
| Colson 2013 | **Acceptance of treatment for latent tuberculosis infection: prospective cohort study in the United States and Canada.** | Wrong age group |
| Daskalaki 2011 | **Tolerability of rifampin monotherapy for latent tuberculosis infection in children.** | Letter to the Editor |
| Denholm 2017 | **SIRCLE: a randomised controlled cost comparison of self-administered short-course isoniazid and rifapentine for cost-effective latent tuberculosis eradication.** | Wrong age group |
| Eidlitz-Markus 2003 | **Use of the urine color test to monitor compliance with isoniazid treatment of latent tuberculosis infection** | Single regimen assessment |
| Erkens 2016 | **Monitoring latent tuberculosis infection diagnosis and management in the Netherlands** | Mixed outcomes for different cohorts |
| Esfahani 2011 | **Potential cost-effectiveness of rifampin vs. isoniazid for latent tuberculosis: implications for future clinical trials** | Wrong age group |
| Feng 2023 | **Trends in tuberculosis clinicians' adoption of short-course regimens for latent tuberculosis infection** | Wrong outcomes: trends of therapy adoption |
| Fluegge 2014 | **Assessing the impact of patient self-selection on the costs to treat latent tuberculosis infection (LTBI) with isoniazid and transitional rifampin.** | Wrong age group |
| Froberg 2019 | **Treatment of latent tuberculosis with 12 weeks isoniazid/rifapentine in clinical practice** | Single regimen assessment |
| Gonzalez-Reyes 2024 | **Interpreter usage and associations with latent tuberculosis infection treatment acceptance and completion in the USA among non-U.S.-born persons, 2012-2017** | Wrong outcomes: LTBI treatment acceptance |
| Gray 2016 | **Baseline abnormal liver function tests are more important than age in the development of isoniazid-induced hepatoxicity for patients receiving preventive therapy for latent tuberculosis infection.** | Mixed data for children and adults |
| Gullon-Blanco 2021 | **Completion of treatment for latent TB infection in a low prevalence setting** | Letter to the Editor |
| Horsburgh 2010 | **Latent TB infection treatment acceptance and completion in the United States and Canada.** | Wrong comparator: no data on shorter regimens |
| Huang 2016 | **Impacts of 12-dose regimen for latent tuberculosis infection: Treatment completion rate and cost-effectiveness in Taiwan.** | Mixed data for children and adults |
| Huang 2020 | **Isoniazid preventive therapy in contacts of multidrug-resistant tuberculosis** | Single regimen assessment |
| Jung 2024 | **Adverse drug reactions following treatment of latent tuberculosis infection: a linked national tuberculosis surveillance with claims database** | Mixed data for children and adults |
| Kawatsu 2021 | **Trend and treatment outcomes of latent tuberculosis infection among migrant persons in Japan: retrospective analysis of Japan tuberculosis surveillance data** | Mixed outcomes for different cohorts |
| Kim 2021 | **Latent Tuberculosis Infection Screening and Treatment in Congregate Settings (TB FREE COREA): Demographic Profiles of Interferon-Gamma Release Assay Cohort** | Mixed data for children and adults |
| Lardizabal 2006 | **Enhancement of treatment completion for latent tuberculosis infection with 4 months of rifampin** | Wrong age group |
| Law 2021 | **Management of latent TB infection in child household contacts aged under 5 years** | Letter to the Editor |
| Lee 2012 | **Latent infection treatment to prevent TB transmission in school settings** | Commentary |
| Li 2010 | **Adherence to treatment of latent tuberculosis infection in a clinical population in New York City.** | Wrong comparator: mixed data for 6-9 and 9-12 months of isoniazid |
| LoBue 2003 | **Use of isoniazid for latent tuberculosis infection in a public health clinic** | Single regimen assessment |
| Macaraig 2018 | **Improved treatment completion with shorter treatment regimens for latent tuberculous infection** | Mixed data for children and adults |
| Martinez-Roig 2003 | **Compliance with tuberculostatic therapy among patients seen at Hospital del Mar in Barcelona. Ten-year follow-up** | Treatment of active TB |
| Martins 2025 | **Rates of Treatment Discontinuation over Time with Rifampin versus Isoniazid for Latent Tuberculosis Infection: A 6-year Experience at a Large Safety-Net Clinic** | Conference abstract |
| McClintock 2017 | **Treatment completion for latent tuberculosis infection: a retrospective cohort study comparing 9 months of isoniazid, 4 months of rifampin and 3 months of isoniazid and rifapentine** | Wrong age group |
| Menzies 2008 | **Adverse events with 4 months of rifampin therapy or 9 months of isoniazid therapy for latent tuberculosis infection: a randomized trial.** | Wrong age group |
| NCT00023452 2001 | **Three Months of Weekly Rifapentine and Isoniazid for M. Tuberculosis Infection** | Study protocol |
| NCT00170209 2005 | **Rifampin Versus Isoniazid for the Treatment of Latent Tuberculosis Infection in Children (P4v9)** | Study protocol |
| NCT00931736 2009 | **Randomized Clinical Trial Comparing 4RIF vs. 9INH for LTBI Treatment-effectiveness** | Study protocol |
| NCT02208427 2014 | **Toward a Safe and Reachable Preventive Therapy for LTBI: a Multicenter Randomized Controlled Study in Taiwan** | Study protocol |
| NCT02613169 2015 | **Infant TB Infection Prevention Study** | Study protocol |
| NCT04094012 2019 | **Risk of SDRs Under 3HP and 1HP Regimen for LTBI** | Study protocol |
| NCT04156568 2019 | **A Multicentre, Cohort Study of Screening and Preventive Intervention for Latent Tuberculosis Infection in Children** | Study protocol |
| Neuhann 2020 | **Contact Investigation in Active Tuberculosis and Management of Latent TB Infection: 5-year Analysis at a German City Health Authority** | Wrong language |
| Oh 2022 | **Four months of rifampicin monotherapy for latent tuberculosis infection in children** | Wrong study design: review article |
| Oh 2025 | **Adverse events of tuberculosis preventive therapy among individuals with latent tuberculosis infection: A nationwide cohort study in South Korea.** | Wrong age group |
| Ohd 2021 | **Evaluation of the latent tuberculosis screening and treatment strategy for asylum seekers in Stockholm, Sweden 2015-2018: a record linkage study of the care cascade** | Wrong comparator |
| Oxlade 2021 | **Effectiveness and cost-effectiveness of a health systems intervention for latent tuberculosis infection management (ACT4): a cluster-randomised trial** | Health systems interventions |
| Page 2006 | **Improved adherence and less toxicity with rifampin vs isoniazid for treatment of latent tuberculosis: a retrospective study.** | Mixed data for children and adults |
| Pease 2019 | **The latent tuberculosis infection cascade of care in Iqaluit, Nunavut, 2012-2016** | Single regimen assessment |
| Pina 2012 | **Is isoniazid for 6 months more cost-effective than isoniazid for 9 months?** | Wrong age group |
| Pina-Gutierrez 2008 | **Adherence and effectiveness of the treatment of latent tuberculosis infection with isoniazid for 9 months in a cohort of 755 patients** | Wrong language |
| Portilla 2003 | **[Directly observed treatment of latent tuberculosis infection: comparative study of two isoniazid regimens].** | Wrong language |
| Rennert-May 2016 | **A Step toward Tuberculosis Elimination in a Low-Incidence Country: Successful Diagnosis and Treatment of Latent Tuberculosis Infection in a Refugee Clinic.** | Mixed outcomes for different cohorts |
| Sadatsafavi 2013 | **A quantitative benefit-risk analysis of isoniazid for treatment of latent tuberculosis infection using incremental benefit framework.** | Wrong study design: hypothetical analysis |
| Sandul 2017 | **High Rate of Treatment Completion in Program Settings with 12-Dose Weekly Isoniazid and Rifapentine for Latent Mycobacterium tuberculosis Infection** | Single regimen assessment |
| Schein 2018 | **Treatment completion for latent tuberculosis infection in Norway: A prospective cohort study** | Wrong comparator: no data on longer regimens |
| Seraphin 2019 | **Timing of treatment interruption among latently infected tuberculosis cases treated with a nine-month course of daily isoniazid: findings from a time to event analysis** | Single regimen assessment |
| Souza 2021 | **Determinants of losses in the latent tuberculosis infection cascade of care in Brazil** | Mixed data for children and adults |
| Spruijt 2019 | **Latent tuberculosis screening and treatment among asylum seekers: A mixed-methods study** | Single regimen assessment |
| Spruijt 2019 | **Implementation of latent tuberculosis infection screening and treatment among newly arriving immigrants in the Netherlands: A mixed methods pilot evaluation.** | Single regimen assessment |
| Sterling 2015 | **Flu-like and Other Systemic Drug Reactions among Persons Receiving Weekly Rifapentine Plus Isoniazid or Daily Isoniazid for Treatment of Latent Tuberculosis Infection in the PREVENT Tuberculosis Study** | Wrong age group |
| Sun 2018 | **Twelve-dose weekly rifapentine plus isoniazid for latent tuberculosis infection: A multicentre randomised controlled trial in Taiwan.** | Mixed data for children and adults |
| Tersigni 2018 | **Latent tuberculosis in childhood: tolerability of two different therapeutic approaches** | Wrong comparator: mixed data for 6 and 9 months of isoniazid |
| Trajman 2010 | **Factors associated with treatment adherence in a randomised trial of latent tuberculosis infection treatment.** | Wrong age group |
| Turkkani 2020 | **The treatment of latent tuberculosis infection: Province based practice and problems** | Single regimen assessment |
| VanHest 2004 | **Hepatotoxicity of rifampin-pyrazinamide and isoniazid preventive therapy and tuberculosis treatment** | Wrong age group |
| Venturini 2018 | **Latent tuberculosis treatment compliance in children: A 10-year experience** | Letter to the Editor |
| Wang 2022 | **US Postarrival Evaluation of Immigrant and Refugee Children with Latent Tuberculosis Infection Diagnosed Overseas, 2007-2019** | Data for separate regimens is not presented |
| White 2012 | **Isoniazid vs. rifampin for latent tuberculosis infection in jail inmates: toxicity and adherence** | Wrong age group |
| Wysocki 2016 | **Latent tuberculosis infection diagnostic and treatment cascade among contacts in primary health care in a city of sao paulo state, Brazil: Cross-Sectional study** | Single regimen assessment |
| Xin 2022 | **Protective efficacy of 6-week regimen for latent tuberculosis infection treatment in rural China: 5-year follow-up of a randomised controlled trial** | Wrong age group |
| Yang 2021 | **High rate of completion for weekly rifapentine plus isoniazid treatment in Chinese children with latent tuberculosis infection-A single center study** | Single regimen assessment |
| Young 2012 | **Successful treatment of pediatric latent tuberculosis infection in a community health center clinic** | Single regimen assessment |
| - | **Impact of Nursing Interventions on Adherence to Treatment With Anti-tuberculosis Drugs in Children and AdolescentsA1 - Anonymous.** | Study protocol |
| - | **Promoting Adherence to TB Regimens in Latino AdolescentsA1 - Anonymous.** | Study protocol |
| - | **Acceptability and Completion Rates of a New 12 Dose Treatment (3 Month) Compared to the Standard Treatment for Latent TB Infection TreatmentA1 - Anonymous.** | Study protocol |
| - | **Toward a Safe and Reachable Preventive Therapy for LTBI: a Multicenter Randomized Controlled Study in TaiwanA1 - Anonymous.** | Study protocol |
| - | **TBTC Study 26: Effectiveness and Tolerability of Weekly Rifapentine/Isoniazid for 3 Months Versus Daily Isoniazid for 9 Months for the Treatment of Latent Tuberculosis InfectionA1 - Anonymous.** | Study protocol |
| - | **TBTC Study 26 PK: Rifapentine Pharmacokinetics in Children Receiving Once Weekly Rifapentine and Isoniazid for the Treatment of Latent Tuberculosis InfectionA1 - Anonymous.** | Study protocol |
| - | **Tuberculosis en Ninos: Implementacion de Una Estrategia de Cuidado Integral Para Los Ninos Contacto de Pacientes Con Tuberculosis Pulmonar BaciliferaA1 - Anonymous.** | Study protocol |
| - | **Multicenter Italian Observational Cohort Study on Tuberculosis in Pediatric AgeA1 - Anonymous.** | Study protocol |

**Table S1.** Reasons for study exclusion during full-text screening stage.

| **First author, Year** | **T. Diallo, 2018** | | | | | **N. Spyridis, 2007** | | | | **M. Villarino, 2015** | | | | | **G. Lemvik. 2025** | | | | | | |
| --- | --- | --- | --- | --- | --- | --- | --- | --- | --- | --- | --- | --- | --- | --- | --- | --- | --- | --- | --- | --- | --- |
| **LTBI therapy** | **4 rifampin** | | | **9 INH** | | **4 rifampin + INH** | | **9 INH** | | **3 rifapentine + INH** | | | **9 INH** | | **4 rifampicin + INH** | | | **9 INH** | | | |
| **N of participants/  AEs** | **N** | | **Total** | **N** | **Total** | **N** | **Total** | **N** | **Total** | **N** | **Total** | | **N** | **Total** | **N** | **Total** | | **N** | | | **Total** |
| AEs resulted in therapy discontinuation | 0 | 422 | | 0 | 407 | 0 | 238 | 0 | 232 | 8 | | 471 | 2 | 434 | 1 | | 393 | | 1 | 350 | |
| SERIOUS ADVERSE EVENTS AND DEATH | | | | | | | | | | | | | | | | | | | | | |
| Death, attributed to a trial drug | 0 | 422 | | 0 | 407 | 0 | 238 | 0 | 232 | 0 | | 471 | 0 | 434 | 0 | | 393 | | 0 | 350 | |
| Death, not attributed to a trial drug | 1 | 422 | | 0 | 407 | 0 | 238 | 0 | 232 | 0 | | 471 | 2 | 434 | 0 | | 393 | | 1 | 350 | |
| Serious AEs/grade 3-5 AEs attributed to treatment | 0 | 422 | | 0 | 407 | 0 | 238 | 0 | 232 | 3^a^ | | 471 | 1^b^ | 434 | 0 | | 393 | | 0 | 350 | |
| Serious AEs/grade 3-5 AEs not attributed to treatment |  |  | |  |  |  |  |  |  | 4^c^ | | 471 | 9^d^ | 434 | 5 | | 393 | | 14 | 350 | |
| OTHER ADVERSE EVENTS | | | | | | | | | | | | | | | | | | | | | |
| Grades 1 and 2 attributed to treatment | 0 | 422 | | 0 | 407 |  |  |  |  | 11 | | 471 | 5 | 434 |  | |  | |  |  | |
| Any minor symptom | 46^e^ | 422 | | 77^e^ | 407 |  |  |  |  |  | |  |  |  | 95^f^ | | 393 | | 163^f^ | 350 | |
| Cutaneous events |  |  | |  |  | 14 | 650 | 0 | 200 | 3 | | 471 | 1 | 434 |  | |  | |  |  | |
| Gastrointestinal events |  |  | |  |  | 7 | 238 | 13 | 200 | 2 | | 471 | 1 | 434 |  | |  | |  |  | |
| Transient increase in liver enzymes |  |  | |  |  | 8^g^ | 650 | 12^g^ | 200 | 0 | | 471 | 0 | 434 | 1^h^ | | 393 | | 0^h^ | 350 | |

**Table S2.** Adverse events reported across trials of tuberculosis infection treatment.

Gray-filled cells represent adverse events not reported in a trial.

a – toxicity grade 3: 1 influenza-like event and 2 cutaneous events.

b – toxicity grade 3: hepatomegaly and rash.

c – three events toxicity grade 3 and one event toxicity grade 4.

d – five events toxicity grade 3, two events toxicity grade 4, and two events toxicity grade 5.

e – minor symptoms included fever or night sweats, weight loss, sputum, cough, skin problems, gastrointestinal problems, and neurologic problems.

f –the most commonly reported symptoms were fever and cough.

g – transient increase in liver enzyme levels <3 times the upper limit of normal.

h – transient increase in liver enzyme levels >3 times the upper limit of normal.
