# Supplementary figures and images for: Shorter Antitubercular Regimens Versus 9 Months of Isoniazid for Latent Tuberculosis in Children: A Systematic Review and Meta-Analysis
Source: Clin Infect Dis. 2026 Mar 10;83(1):e155–66. doi: 10.1093/cid/ciag073 (PMC13393115; doi:10.1093/cid/ciag073)

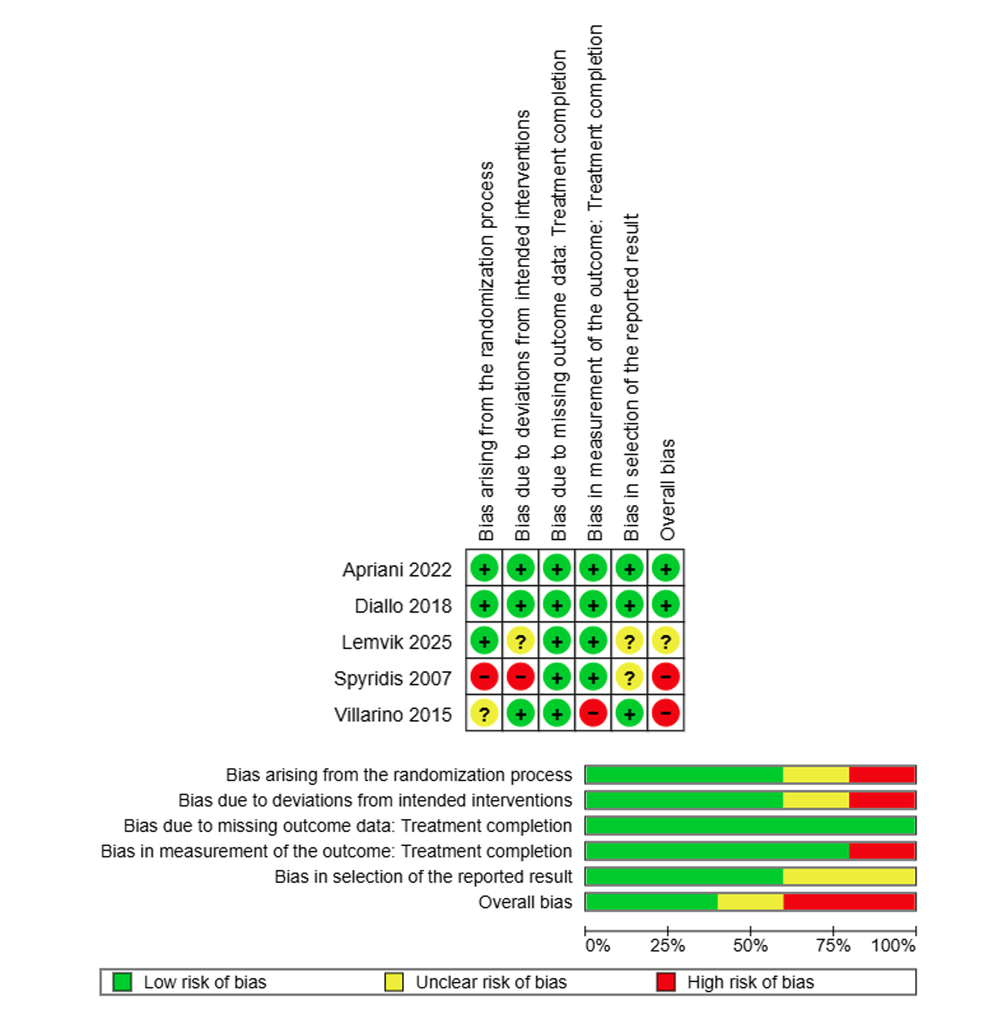

Supplement: ciag073_Supplementary_Data [file ciag073_supplementary_data.zip › figS1_1col_300dpi.tif]

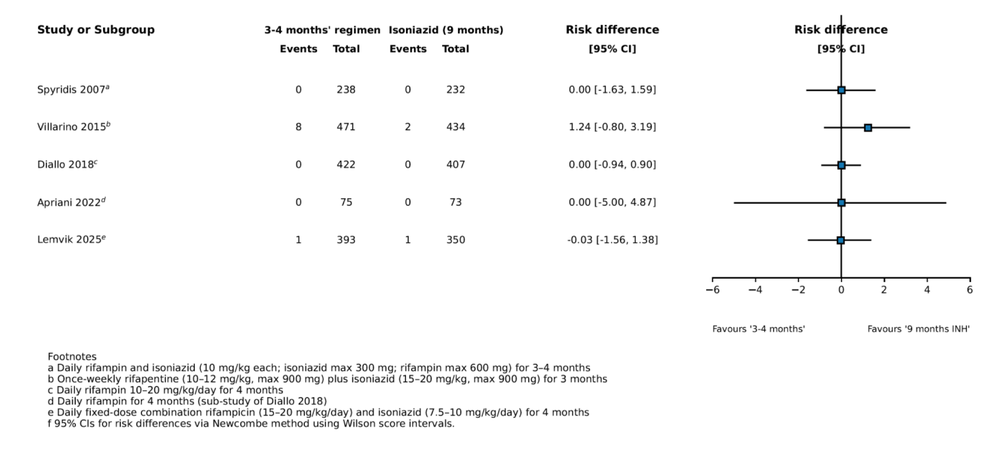

Supplement: ciag073_Supplementary_Data [file ciag073_supplementary_data.zip › figS2_1col_300dpi.tif]
